# The association between the ratio of monocytes:lymphocytes at age 3 months and risk of tuberculosis (TB) in the first two years of life

**DOI:** 10.1186/s12916-014-0120-7

**Published:** 2014-07-17

**Authors:** Vivek Naranbhai, Soyeon Kim, Helen Fletcher, Mark F Cotton, Avy Violari, Charles Mitchell, Sharon Nachman, George McSherry, Helen McShane, Adrian VS Hill, Shabir A Madhi

**Affiliations:** 1 Wellcome Trust Centre for Human Genetics, Nuffield Department of Medicine, University of Oxford, Oxford, UK; 2 Center for the AIDS Program of Research in South Africa (CAPRISA), University of KwaZulu Natal, Durban, South Africa; 3 Center for Biostatistics in AIDS Research, Department of Biostatistics, Harvard School of Public Health, Boston, USA; 4 The Jenner Institute, Nuffield Department of Medicine, University of Oxford, Oxford, UK; 5 Children’s Infectious Diseases Clinical Research Unit, Stellenbosch University, Stellenbosch, South Africa; 6 The Perinatal HIV Research Unit, University of the Witwatersrand, Johannesburg, South Africa; 7 University of Miami, Florida, USA; 8 Department of Pediatrics, State University of New York at Stony Brook, Stony Brook, USA; 9 Department of Pediatrics, Pennsylvania State University College of Medicine, Hershey, Pennsylvania, USA; 10 Medical Research Council: Respiratory and Meningeal Pathogens Research Unit, University of Witwatersrand, Johannesburg, South Africa

**Keywords:** Tuberculosis, HIV, combination antiretroviral therapy, monocytes, lymphocytes, ML ratio

## Abstract

**Background:**

Recent transcriptomic studies revived a hypothesis suggested by historical studies in rabbits that the ratio of peripheral blood monocytes to lymphocytes (ML) is associated with risk of tuberculosis (TB) disease. Recent data confirmed the hypothesis in cattle and in adults infected with HIV.

**Methods:**

We tested this hypothesis in 1,336 infants (540 HIV-infected, 796 HIV-exposed, uninfected (HEU)) prospectively followed in a randomized controlled trial of isoniazid prophylaxis in Southern Africa, the IMPAACT P1041 study. We modeled the relationship between ML ratio at enrollment (91 to 120 days after birth) and TB disease or death in HIV-infected children and latent *Mycobacterium tuberculosis* (MTB) infection, TB disease or death in HEU children within 96 weeks (with 12 week window) of randomization. Infants were followed-up prospectively and routinely assessed for MTB exposure and outcomes. Cox proportional hazards models allowing for non-linear associations were used; in all cases linear models were the most parsimonious.

**Results:**

Increasing ML ratio at baseline was significantly associated with TB disease/death within two years (adjusted hazard ratio (HR) 1.17 per unit increase in ML ratio; 95% confidence interval (CI) 1.01 to 1.34; *P* = 0.03). Neither monocyte count nor lymphocyte counts alone were associated with TB disease. The association was not statistically dissimilar between HIV infected and HEU children. Baseline ML ratio was associated with composite endpoints of TB disease and death and/or TB infection. It was strongest when restricted to probable and definite TB disease (HR 1.50; 95% CI 1.19 to 1.89; *P* = 0.006). Therefore, per 0.1 unit increase in the ML ratio at three to four months of age, the hazard of probable or definite TB disease before two years was increased by roughly 4% (95% CI 1.7% to 6.6%).

**Conclusion:**

Elevated ML ratio at three- to four-months old is associated with increased hazards of TB disease before two years among children in Southern Africa. While significant, the modest effect size suggests that the ML ratio plays a modest role in predicting TB disease-free survival; its utility may, therefore, be limited to combination with existing tools to stratify TB risk, or to inform underlying pathophysiologic determinants of TB disease.

## Background

Globally, *Mycobacterium tuberculosis* (MTB) infects about 2 billion people, causing 10 million active cases, of whom about 500,000 cases are children [[1]]. It is a leading cause of death in sub-Saharan Africa, yet practical methods to stratify risk in this population are lacking [[2],[3]]. Children with MTB infection are at risk for tuberculosis (TB) disease; therefore, identification of these children is important. The current tools to identify children with MTB infection are tuberculin skin testing (TST) or interferon-gamma release assay (IGRA). Although isoniazid (INH) preventive therapy (IPT) is more effective in HIV-infected individuals with a positive TST result [[4]], neither TST nor IGRA are sufficiently good at predicting TB disease. In a recent meta-analysis, a positive TST or IGRA result in children or adults was associated with an increase in incidence of about 1.4 to 2 fold with wide confidence limits due to study heterogeneity [[3]]. Current World Health Organization (WHO) guidelines do not support routine TST or IGRA testing before IPT provision in children [[5]]. If individuals at risk for TB could be accurately identified, they could be targeted for interventions to prevent TB such as IPT. In addition, predictors of TB may offer new insight into pathogenesis.

Recent and historical studies suggest that the peripheral blood monocytes:lymphocytes ratio may be associated with subsequent mycobacterial disease outcomes. Fletcher *et al*. used whole-transcriptome microarrays to examine leucocyte gene expression in 10-week-old BCG-vaccinated, HIV-uninfected infants for clues as to why some developed TB by age two years while others did not [[6]]. They found that opposing levels of myeloid and lymphoid specific gene transcripts were more frequent among infants who later developed culture-confirmed TB disease, than among matched controls. Since the quantity of myeloid and lymphoid transcripts in peripheral blood is a marker of the frequency of leucocyte subsets, the transcript ratio may reflect leucocyte subset frequencies. However, these were not available in their study. Nevertheless, in support of the specific role of relative proportions of monocytes and lymphocytes in TB pathogenesis, studies performed between 1921 and 1930 by Florence Sabin and colleagues demonstrated in rabbit models of TB that ‘monocyte-lymphocyte ratio in the peripheral blood might be taken as an index of the progress and extent of the disease’ [[7]]. Sabin and colleagues then demonstrated that experimentally altering the ratio by depleting or increasing monocyte frequency resulted in commensurate changes in rabbit survival following challenge with *Mycobacterium bovis* [[7]–[9]]. Recent results in cattle show *in vitro* that the ratio of monocyte-derived macrophages to lymphocytes is associated with inhibition of mycobacterial growth [[10],[11]]. We recently reported that the ratio of monocytes:lymphocytes (ML) in HIV-infected South African adults prior to combination antiretroviral therapy initiation (cART) was predictive of TB disease during the subsequent five years on cART [[12]]. In what appears to be the first and only study of the role of this ratio in childhood mycobacterial disease in 1928, Rogers studied the ML ratio in 41 children with either a negative tuberculin skin test, latent TB infection or active TB disease. He observed that the ML ratio correlated with the extent and severity of disease, but called for ‘the work [to] be checked up on a larger series’ [[13]].

Here, we evaluated whether the peripheral blood ML ratio at between three- and four- months of age was associated with subsequent MTB infection or TB disease during the first two years of life in infants randomized to either INH prophylaxis or placebo in the IMPAACT P1041 study [[14]].

## Methods

### Study population

In IMPAACT P1041 (clinicaltrials.gov reference NCT00080119), 544 HIV-infected and 810 HIV-exposed, uninfected infants (HEU), 91 to 120 days of age, born to HIV-infected mothers, were enrolled at one of three sites in South Africa (Chris Hani Baragwanath Hospital, Johannesburg; Tygerberg Hospital, Stellenbosch University, Cape Town, and King Edward VIII Hospital, Durban) or at one center in Botswana (Princess Marina Hospital, Gaborone). Infants were randomized to receive INH 10 to 20 mg per kilogram body weight daily or placebo. Results of the trial have been reported [[14]]. Informed consent for participation in this study, specimen collection and future research was obtained from parents/guardians under protocols approved at each study site (University of the Witwatersrand: #020109; Stellenbosch University: #M04/05/026; and University of KwaZulu Natal: #T118/05) and by the Oxford Tropical Research Ethics Committee (Reference 508–13).

### Definition of ML ratio

Investigations in P1041 included an enrollment full blood count, including white blood cell differential count, which was repeated three-monthly thereafter. Full blood counts were obtained using routine flow-cytometric assays in accredited laboratories.

### Study endpoints

The primary outcome measure of this study was TB disease or death in HIV-infected children and latent MTB infection, TB disease or death in HEU children within 96 weeks (with 12 week window) after randomization. Infants were followed-up prospectively and routinely assessed for MTB exposure and outcomes as reported [[14]]. An endpoint review committee of study-team clinicians unaware of the study-group assignments reviewed all deaths and potential TB-related primary and secondary endpoints. Latent MTB infection among HIV-uninfected infants was diagnosed by TST at approximately two years of age (and again at three and four years for a subset), or when clinically indicated. Latent MTB infection was defined as a positive TST (induration ≥5 mm in horizontal diameter in HIV-infected children and ≥10 mm in HEU children) in the absence of evidence of active disease according to contemporary guidelines [[15]]. The criteria used for categorizing TB as either possible, probable, or microbiologically confirmed (definite) have been previously detailed [[14]].

### Statistical methods

To calculate the ML ratio, the absolute monocyte count was divided by the absolute lymphocyte count. Analysis was performed according to a statistical analysis plan developed *a priori.* To model the association between ML ratio and the primary endpoint, Cox proportional hazards models were used. Scaled Schoenfeld residuals were inspected to evaluate the proportionality assumptions of the Cox model. Study duration was calculated as the time from randomization to the first diagnosed episode of TB or death, withdrawal from the study or the end of week 108 (last visit date for patients remaining in follow-up), whichever occurred first. Where possible we preferred modeling continuous variables as fractional polynomials [[16],[17]], an approach that allows continuous variables to be analyzed on their native scale without categorization, and allowing for non-linear fits. We explored non-linear fits with fractional polynomials, in all cases the best statistical fit was linear.

All statistical tests were two-sided at the 5% significance level. Poisson approximations were used to calculate confidence intervals (CIs) for estimations of the incidence rate. Bootstrapped estimates of the adjusted hazard ratio (HR) across the ML ratio continuum were generated with the ‘*boot*’ package. Statistical analyses were performed in R (The R Foundation for Statistical Computing) using the following packages ‘epiR’, ‘survival’, ‘date’ and ‘mfp’.

## Results

Between December 2004 and June 2008, 1,354 infants born to HIV-infected mothers were enrolled at a median of 96 days of age (interquartile range (IQR) 92 to 105 days). Their baseline characteristics have been reported [[14]]. A total of 1,336 infants were eligible for this analysis. The reasons for ineligibility were a missing full blood count result (nine participants), implausible results (two participants), participant not receiving INH/placebo and not followed (three participants) or missing covariates or outcomes (four participants). The included infants were similar to those in the primary analysis [[14]]: 47.8% (638/1,336) were male, 25.2% (136/540) of all HIV-infected infants were receiving cART at baseline, 7.2% (96/1,336) had a maternal history of TB, and 9% (121/1,215) were ever breastfed. The median WHO weight for age z-score was −0.55 (IQR −1.45 to 0.28).

**Figure 1:**
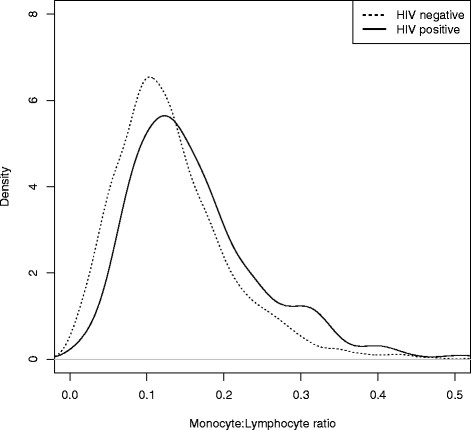
Distribution of monocyte:lymphocyte (ML) ratio at enrollment shown for HEU (n = 796, dashed line) and HIV-positive infants (n = 540, solid line).

The median ML ratio at baseline was 0.13 (IQR 0.09 to 0.18). As shown in Figure 1, the distribution of ML ratio in HIV-infected infants was significantly higher (median 0.15 versus 0.12, *P* <0.001) than HEU infants. This was consistent with the higher absolute monocyte counts among HIV-infected infants (median 0.93 versus 0.71, *P* <0.001) but similar absolute lymphocyte counts (6.02 versus 5.89, *P* = 0.15 ) compared to HEU infants.

### Baseline ML ratio is associated with TB disease free survival

Over 1,997 infant-years, 187 infants reached the primary endpoint of this study: TB disease (probable, possible or definite) or death in HIV-infected infants and latent MTB infection, TB disease or death in HEU infants. Stratified by HIV status, the unadjusted HR per 1 unit increase in the ML ratio was 1.20 (95% CI 1.05 to 1.38, *P* = 0.008). Adjusting for the weight-for-age z-score, receipt of cART at the time of sampling, a maternal history of TB, breastfeeding and receipt of INH prophylaxis, did not markedly alter the effect estimate (HR 1.17 (1.01 to 1.34), *P* = 0.03). In contrast, neither the monocyte nor lymphocyte count alone was significantly associated with the primary study endpoint. This finding is consistent with previous studies in adult humans [[12]], in rabbits [[9]] and *in vitro* (data not shown). Adjusting for CD4% among HIV-infected infants did not weaken the association in this group (HR 1.2; 95% CI: 1.01 to 1.43, *P* = 0.04), nor did adjusting for absolute leucocyte counts in the entire cohort (HR 1.20; 95% CI: 1.01 to 1.43).

### Baseline ML ratio is associated with probable or definite TB rather than all-cause mortality or latent MTB infection

To explore whether the ML ratio was specifically associated with TB disease or with other outcomes, we tested whether the association between the ML ratio and TB disease was robust to alternative endpoint definitions (Table 1). The ML ratio was significantly associated with elevated hazards of TB disease alone (HR 1.23, 95% CI 1.04 to 1.45, *P* = 0.02) and with elevated hazards of TB disease plus all-cause death (HR 1.18, 95% CI 1.02 to 1.37, *P* = 0.02) or TB disease plus latent MTB infection (HR1.21, 95% CI 1.04 to 1.41, *P* = 0.02). Increasing the stringency of the case definition by excluding individuals with possible TB and including only definite and probable TB diagnoses strengthened the association between ML ratio and TB disease (HR 1.50, 1.19 to 1.89, *P* = 0.006). The ML ratio was not significantly associated with all-cause death (HR 1.25, 95% CI 0.99 to 1.57, *P* = 0.06). Among HEU children, with TST at 96 weeks, 33 of 725 (4.5%) had latent MTB infection. There was no association between baseline ML ratio and latent MTB infection (HR 1.00, 95% CI 0.66 to 1.5, *P* = 0.99) neither was there evidence of interaction between ML ratio and INH prophylaxis. No INH effect was observable in a model of INH effect across the ML ratio spectrum.

**Table 1:** Sensitivity analysis: the association between baseline ML ratio and TB outcomes holds across TB outcome grouping

**Endpoint**	**Number (cases/at risk)**	**Multivariable regression adjusted hazard ratio (95% CI)**^**a**^	***P*** **value**
Primary endpoint: TB disease^b^ or death in HIV + infants; and TB disease^b^, death or latent MTB infection in HEU infants	187/1336	**1.17 (1.01** to **1.34)**	**0.03**
TB disease^b^ or death	166^c^/1336	**1.18 (1.02** to **1.37)**	**0.02**
TB disease^b^ or latent MTB infection	152/1336	**1.21 (1.04** to **1.41)**	**0.02**
TB disease^b^	126/1336	**1.23 (1.04** to **1.45)**	**0.02**
Probable and definite TB only	49/1336	**1.50 (1.19** to **1.89)**	**0.006**
Death	50/1336	1.25 (0.99 to 1.57)	0.06
Latent MTB infection^d^	33/725	1.00 (0.66 to 1.5)	0.99

^a^Estimated from Cox Proportional Hazard regression models stratified by infant HIV status, modeling ML as linear variable following fractional polynomial exploration, adjusted for weight-for-age z-score, cART receipt at baseline, maternal history of TB, ever breastfed and randomized INH prophylaxis/placebo group. Hazard ratios are per unit increase in the ML ratio. ^b^Possible, probable or definite TB. ^c^Participants who had TB disease (possible, probable or definite TB) and subsequently died were only counted once, hence this number is not the sum of the TB disease and death numerators below. ^d^Latent MTB infection was only assessed in HEU infants. Text in bold highlights statistically significant findings. cART, combination antiretroviral therapy; CI, confidence interval; HEU, HIV-exposed uninfected; INH, isoniazid; MTB, *Mycobacterium tuberculosis*; TB, tuberculosis.

### The association between baseline ML ratio and TB disease free survival is similar in HIV-infected and HEU children

The association between ML ratio and the primary endpoint was not statistically dissimilar between HIV-infected and HEU infants (adjusted HR 1.2, 95% CI 1.01 to 1.42 versus adjusted HR 1.07, 95% CI 0.83 to 1.4 respectively; Chi^2^ test for heterogeneity with 1df: *P* = 0.48, overall test for effect *P* = 0.04).

## Discussion

Transcriptomic studies in infants suggested that the relative proportions of myeloid and lymphoid cells in infants have predictive value for TB risk (HF, unpublished). Concordant animal studies support this notion [[9]], as do recent studies in adults with AIDS [[18]]. We found that the ML ratio in peripheral blood at around three months of age was associated with TB disease or MTB infection-free survival in South African children, notwithstanding conventional risk factors. Per 0.1 unit increase in ML ratio at three months of age, the hazard of probable or definite TB disease before two years old increased by roughly 4% (approximately 1.5^0.1^). While significant, the modest effect size suggests that the ML ratio plays a modest role in predicting TB disease-free survival; its utility may, therefore, be limited to combination with other tools to stratify TB risk or to study pathophysiologic determinants of TB disease.

Several factors support the ML ratio being on the causal pathway for TB development. First, monocytes are target cells for mycobacterial growth and lymphocytes are the major effectors for mycobacterial clearance. Second, there appears to be a dose or gradient effect with higher ratios being more predictive than lower ratios across the ML ratio gradient. Third, as we demonstrated, altered ML ratios precede active disease; hence, reverse causality is unlikely. Fourth, our data in children are consistent with *in vitro* findings. Fifth, there is overall coherence of this finding with experimental animal and observational adult studies. Finally, the association between the ML ratio and subsequent TB disease has partial specificity. The association is stronger for the ML ratio rather than monocyte counts and definite/probable TB rather than possible TB. We have also recently reported that the ML ratio may be associated with childhood malaria incidence [[19]]; hence, the ML ratio may actually have pleiotropic associations with some childhood infectious diseases. However, the effect size seems modest. Further studies should endeavor to assess more detailed subsets of monocytes and lymphocytes to identify precise cellular players in this association.

The mechanistic basis for the association is not addressed in this work. It is plausible that the ML ratio reflects relative frequency of myeloid or lymphoid biased hematopoietic stem cells, and that the ratio may, therefore, reflect ontogeny driven differences in function [[20],[21]]. This argument remains speculative. Alternatively, the ML ratio may reflect the relative frequency of monocytes as target cells and lymphocytes as effectors against TB. The likelihood of the latter explanation is reduced by the lack of association between monocyte counts and/or lymphocyte counts alone with TB outcomes. A third possible explanation is that an altered ML ratio is due to a specific monocyte subset defect. Since HIV infects and alters monocyte function in a subset specific manner [[22]], it is possible that HIV exposure alters monocyte functions and ratios. The similarity in effect between HIV-infected and HEU infants, however, mitigates the likelihood of the association being driven by HIV infection.

Our analysis has several limitations. Firstly, the definition of TB outcomes is challenging in infants. This analysis benefits from using data from a trial where outcomes were stringently defined and prospectively recorded, without knowledge of this hypothesis. Clearly specified definitions, including those delineating certainty of endpoints, allowed evaluation of the sensitivity of our finding to alternative outcome definitions, including more stringent definitions of TB disease. Secondly, this observational study cannot conclusively demonstrate a causal relationship between the ML ratio and TB disease. Mendelian randomization approaches, leveraging genetic correlates of the ratio, may facilitate more stringent evaluation of the association. Thirdly, although we observed association between the ML ratio and incident TB disease in all HIV-exposed infants regardless of HIV infection status, further studies are required to establish whether the association is present in infants not exposed to HIV. Use of an *a priori* statistical analysis plan ameliorated the likelihood of the significant association between the ML ratios and TB risk being a false discovery due to multiple hypothesis testing.

## Conclusions

Our study extends observations on the ML ratio and risk of TB disease previously seen in HIV-infected adults to HIV-exposed infants [[18]]. The modest effect size suggests that the ML ratio plays a modest role in predicting TB disease risk in infants. Therefore, for clinical utility, the ML ratio may benefit from combination with other tools to identify at-risk infants. The replication of this finding across animal, infant and adult studies nevertheless suggests that the ML ratio is a pathophysiological predictor of tuberculosis disease and further study of this trait may yield insight into why some infants succumb to TB disease.

## Abbreviations

cART: combination antiretroviral therapy

CI: confidence interval

HEU: HIV-exposed, uninfected

HR: hazard ratio

IGRA: interferon gamma release assay

IMPAACT: International Maternal Pediatric Adolescent AIDS Clinical Trials Group

INH: isoniazid

IPT: isoniazid preventive therapy

ML: monocyte:lymphocyte ratio

MTB: *Mycobacterium tuberculosis*

TB: tuberculosis

TST: tuberculin skin testing

WHO: World Health Organization

## Competing interests

The authors declare that they have no competing interests.

## Authors’ contributions

VN and SAM designed the study. VN conceived the idea for this study, conducted the analysis along with SK and wrote the first draft of the manuscript. SAM and AVSH supervised the analysis and conduct of this study. SAM, AV, MC, GM, SN and CM led the clinical trials/studies from which data for this study was obtained. HF and HcM provided critical input on design, analysis and interpretation of the study. All the authors had access to the data. All authors read and approved the final manuscript.
